# Detection of Influenza a Virus in Swine Nasal Swab Samples With a Wash-Free Magnetic Bioassay and a Handheld Giant Magnetoresistance Sensing System

**DOI:** 10.3389/fmicb.2019.01077

**Published:** 2019-05-21

**Authors:** Diqing Su, Kai Wu, Venkatramana D. Krishna, Todd Klein, Jinming Liu, Yinglong Feng, Andres M. Perez, Maxim C.-J. Cheeran, Jian-Ping Wang

**Affiliations:** ^1^Department of Chemical Engineering and Materials Science, University of Minnesota, Minneapolis, MN, United States; ^2^Department of Electrical and Computer Engineering, University of Minnesota, Minneapolis, MN, United States; ^3^Department of Veterinary Population Medicine, College of Veterinary Medicine, University of Minnesota, St. Paul, MN, United States

**Keywords:** influenza A virus, nasal swab sample, wash-free, handheld, magnetic bioassay, giant magnetoresistance, on-site diagnosis

## Abstract

The dissemination of Influenza A virus (IAV) throughout the world has become one of the main concerns for the health of both animals and human beings. An efficient and sensitive diagnostic tool is thus needed for the early detection of IAV. Here, we developed a wash-free magnetic bioassay and further integrated it with a handheld platform based on giant-magnetoresistance (GMR) sensors. The wash-free magnetic bioassay significantly accelerates and simplifies the detection process. This brand-new system was successful in detecting both IAV nucleoprotein and IAV-contained nasal swab samples from pigs on the farm. The limit of detection (LOD) is 0.3 nM for IAV nucleoprotein and 250 TCID_50_/mL for IAV-spiked nasal swab samples. The detection of nasal swab samples containing unpurified IAV was also performed, demonstrating the capability of the magnetic wash-free assay in the detection of biomarkers in complex sample matrix.

## Introduction

Influenza viruses are enveloped, single-strand, negative-sense RNA viruses with segmented RNA genome. Serologically, they are classified into serotype A, B, or C based on differences of their matrix (M) and nucleoprotein (NP) antigens (Deepa, 1971). Influenza A viruses (IAV) are further grouped into subtypes based on their hemagglutinin (HA) and neuraminidase (NA) surface glycoproteins. IAV infects different host species including humans, pigs, and birds. It has been shown that pigs and migratory birds were susceptible to the infection of influenza viruses from human and avian origin and were the main species contributing to the global dissemination of IAVs (Kida et al., 1994; Ren et al., 2016). Therefore, a rapid and sensitive method for the early IAV detection in these species and others is critical to control virus transmission. Currently, there are different laboratory methods available for the diagnosis of IAV infection, including virus isolation in embryonated chicken eggs or cell culture, detection of viral RNA by reverse transcription-quantitative polymerase chain reaction (RT-qPCR), serological tests to detect virus-specific antibodies, and detection of viral antigens by rapid influenza diagnostic tests (RIDTs) such as lateral flow tests and Enzyme linked immunosorbent assays (ELISA) (Lee et al., 1993; Townsend et al., 2006; Leuwerke et al., 2008; Chen et al., 2011). Virus isolation technique is a sensitive but labor-intensive method, which requires considerable experience and takes at least 3 days for the results (Ellis and Zambon, 2002; Amano and Cheng, 2005). RT-qPCR based techniques are the most accurate detection methods, but they can only be performed in laboratories, which is expensive and requires specific expertise (Ellis and Zambon, 2002; Payungporn et al., 2006). Detection of virus-specific antibodies by hemagglutination inhibition assay (HIA) and neutralization tests are also widely used. However, it usually takes 8 to 14 days for antibodies to develop after infection (Miller et al., 2010), which limit the value of this assay during acute phase of infection. Although RIDTs can be performed onsite, they were shown to have a sensitivity of only 50 to 70% in the detection of IAV for RT-PCR positive samples (Chartrand et al., 2012; Merckx et al., 2017). A diagnostic platform that is capable of performing point-of-care tests on influenza viruses with high sensitivity and minimum laboratory skill requirements will have a significant impact on controlling the dissemination of influenza viruses. Recently, a rapid point of care molecular diagnostic test utilizing isothermal nucleic acid amplification technology for the detection of influenza viral RNA in direct nasal swab samples has received FDA-clearance (Wang et al., 2018).

Sensors based on the giant magnetoresistance (GMR) effect are promising candidates for biomarker detection. The first biosensor system based on the GMR effect was proposed by Baselt et al. (1998) The basic structure of GMR sensors are two ferromagnetic layers separated by a metallic layer. It was demonstrated that the multilayer GMR sensors with magnetic microbeads bound to the surface, or the Bead Array Counter (BARC), held a great promise for measuring intermolecular forces during biomolecular recognition. Since then, this idea had been widely used for the detection of biomarkers either with GMR multilayers or spin valve (SV) sensors (Tondra et al., 2000; Zhi et al., 2012; Rizzi et al., 2014; Wang et al., 2015; Dias et al., 2016; Krishna et al., 2016; Cardoso et al., 2017; Wu et al., 2017a). During the detection, the sensor surface is functionalized so that the target antigen could specifically bind to the sensor. By attaching the magnetic nanoparticle (MNP) tags to the target antigens via another layer of antibodies, the stray field generated by the MNPs results in the resistance change of the GMR sensors, which is proportional to the number of captured target antigens. Magnetic Tunnel Junctions (MTJs) have also been used for biological detection recently. The difference between GMR and MTJ sensors are that the two ferromagnetic layers in MTJs are separated by an insulating layer. The complicated fabrication processes, relatively small sensing area, and the existence of top electrodes made MTJs less favorable compared to GMR sensors (Reiss et al., 2005). Furthermore, the GMR sensors also exhibited superior performance such as lower background noise, higher sensitivity and the potential of integration with System on Chip techniques, with respect to other commonly used approaches such as fluorescence sensors (Schotter et al., 2004; Weiss et al., 2013).

In our previous work, we developed a Z-lab handheld platform based on GMR sensors for the detection of IAV (Krishna et al., 2016; Wu et al., 2017a). The device is of the size of a snack container and is capable of detecting real-time GMR sensor signals before and after the addition of analyte. Although the Z-lab was proved to be much more portable and easy-to-use than the benchtop systems, the process of building up the capture antibody-antigen-detection antibody-MNP structure layer by layer involved in multiple washing and incubating steps, which is not only time consuming but also challenging for non-technicians. Furthermore, only the detection of purified virus samples was demonstrated even though the ability to detect IAV in unprocessed samples taken directly from the field is more crucial for the onsite diagnosis. In this paper, we will describe the first wash-free magnetic bioassay with the ability to detect both purified IAV nucleoprotein and IAV in nasal swab samples from pigs. The wash-free bioassay greatly simplifies the testing process compared to the traditional assay preparation techniques, which makes it possible for non-technicians to perform the biomarker detection procedure at different locations. We employed broadly reactive anti-NP antibodies to sense virus in this assay in order to detect all IAV serotypes, irrespective of their HA and NA subtypes.

## Materials and Methods

### Materials

(3-aminopropyl)triethoxysilane (catalog number 440140, also referred to as APTES), Glutaraldehyde solution (catalog number G5882), bovine serum albumin (catalog number A2153, also referred to as BSA), and biotin-conjugated BSA (catalog number A8549) were purchased from Sigma-Aldrich, Inc., USA. InVivoMab anti-influenza A virus NP [catalog number BE0159, clone H16-L10-4R5 (HB-65)] was purchased from Bio X Cell, USA, and used as capture antibody. The recombinant influenza H1N1 nucleoprotein was purchased from Sino Biological Inc, China. Mouse anti-influenza A monoclonal antibody (catalog number MAB8257B) was purchased from EMD Millipore Corporation, USA. Streptavidin functionalized magnetic nanoparticles (2 × 10^12^ particles/mL; Catalog number 130-048-101), also referred to as MACS, were purchased from Miltenyi Biotec, Inc., USA.

### Viruses

Since swine are susceptible to infection with human influenza viruses, representative isolates from both human and swine viruses were used in this study. The human pandemic influenza A/California/04/2009 (pH1N1 CA/09), human influenza A/Victoria/2011 (H3N2 VI/11), influenza A H3N2 variant virus (also known as H3N2v virus) A/Indiana/10/2011 and swine influenza virus A/Swine/Iowa/73 (H1N1 IA/73) were obtained from the University of Minnesota Veterinary Diagnostic Laboratory (St. Paul, MN). Viruses were propagated in Madin-Darby canine kidney (MDCK) cells (ATCC CCL-34) in Dulbecco's modified Eagle medium (DMEM) containing 0.5 μg/mL TPCK-trypsin (Worthington Biochemical Corporation, Lakewood, NJ) and purified by ultracentrifugation through a 30% (w/v) sucrose cushion and stored in aliquots at −80°C. Culture supernatant from un-infected MDCK cells was processed similarly to use for mock virus preparation. The concentration of purified virus was determined by TCID_50_ assay on MDCK cells. For immunoassays, the virus was inactivated at 60°C for 1 h. To disrupt the virus particles, the mock and virus were treated with an equal volume of 1% IGEPAL CA-630 (Sigma-Aldrich, St. Louis, MO) and incubated at 37°C for 10 min.

### Nasal Swab Samples

Nasal swab samples were collected from pigs using BBL Culture swab collection and transport system (Becton Dickinson). Swabs were placed in 1.8 mL of DMEM containing 2% bovine serum albumin (BSA), vortexed for 10 s and stored at −80°C until testing.

### IAV Spiked Samples

Nasal swab samples from 4 IAV-negative pigs (according to RT-PCR) were processed as described above and pooled. For preparing nasal swab samples with different strains of IAV, pooled nasal swab samples were spiked separately with cell culture propagated unpurified pH1N1 CA/09, H1N1 IA/73, and H1N1 VI/01 at 1/1000 dilution. For determining the limit of detection (LOD) of IAV in nasal swab samples, pooled nasal swab samples from IAV negative pigs were spiked with four dilutions of purified pH1N1 CA/09 or purified H3N2v at final concentration of 250, 500, 1,000, and 10,000 TCID_50_/mL.

### GMR Nanosensor Array and Chip Fabrication

GMR stacks with a structure of Ta(50 Å)/NiFe(20 Å)/CoFe(10 Å)/Cu(33 Å)/CoFe(25 Å)/IrMn(80 Å)/Ta(25 Å) were deposited on 4-inch silicon wafers by a six-target magnetron sputtering system (Shamrock). Subsequently, two independent sensor arrays with a total number of 58 sensors were defined on each chip via ion milling, followed by the deposition of Cr(250 Å)/Au(2500 Å)/Cr(150 Å) electrodes. Each sensor with a size of 150 × 100 μm was made up of 24 GMR stripes, which were 150 μm long and 750 nm wide (see Figure S1). To protect the sensors and circuits from the chemicals used in subsequent surface functionalization and biological detection processes, the non-sensing area was passivated with SiO_2_ (5000 Å) using e-beam evaporation, while the sensing area was deposited with Al_2_O_3_ (180 Å) and SiO_2_ (150 Å) by Atomic Layer Deposition (ALD) and Plasma Enhanced Chemical Vapor Deposition (PECVD), respectively. The thinner oxides in the sensing area not only increased the magnetic signal by reducing the distance between the sensor surface and MNPs, but also provided the hydroxyl groups needed for the immobilization of the capture antibody. The fabricated GMR chips were annealed at 200°C for 1 h under a magnetic field of 5 kOe along the short axis of the stripe to align the magnetization direction of the pinned layer. The magnetization direction of the free layer at zero external field was along the long axis of the stripe due to shape anisotropy. A typical magnetoresistance (MR) curve of the GMR sensor was shown in Figure S2.

### Sensor Surface Functionalization

The annealed chips were treated with ultraviolet light and ozone (UVO) for 15 min after sonicated with acetone, methanol, and isopropyl alcohol in sequence. To introduce the aldehyde groups needed for the immobilization of capture antibody, the chips were firstly immersed in 5 mL 1% APTES with anhydrous toluene as the solvent. After 1 h, the surface of the chips was rinsed with acetone and ethanol, and 50 μL 5% glutaraldehyde in DI water was subsequently dropped on each sensor array. The chips were then incubated for 5 h to allow the binding between the aldehyde groups on the glutaraldehyde and the amino groups on APTES. This will result in an excess of aldehyde groups on the sensor surface, which can bind to the amino groups on the capture antibody (see Figure S3). 1.08 nL 1 mg/mL capture antibody was robotically spotted on the surface of each sensor via a programmable liquid dispensing system (sci-FLEXARRAYER S5, Scienion, Germany). The first column and last column of sensors in each sensor array were spotted with 1 mg/mL BSA and 1 mg/mL biotin-BSA, respectively, which served as the control groups (see Figures S4, S5). After incubating at 4°C for 12 h, two bottomless reaction wells with a volume of 50 μL were assembled to each sensor array by polydimethylsiloxane (PDMS). Then, 50 μL of 10 mg/mL BSA was added to each reaction well to block the sensor arrays. The BSA was then washed away with PBST (0.05% tween20 in phosphate buffered saline) after 1 h of incubation. Meanwhile, a mixture of 20 μL IAV NP or IAV contained nasal swab samples, 25 μL MACS, and 5 μL 20 μg/mL detection antibody was prepared and incubated for 1 h. The chip was ready for test and the mixture will be added to the reaction well during the test (Wang et al., 2013).

### Magnetic Tags

The magnetic tags were streptavidin-coated superparamagnetic microbeads with an average hydrodynamic volume of 50 nm (Gaster et al., 2011; Wang et al., 2014; Wu et al., 2017b) (2 × 10^12^ particles/mL; Catalog No. 130-048-101, Miltenyi Biotec, Inc., Auburn, CA, USA), referred to as MACS in this paper. Each MACS microbead consists of smaller nanoparticles (α-Fe_2_O_3_ and Fe_3_O_4_ with an average size of ~8 nm) embedded in a matrix of dextran (Wu et al., 2017b). The superparamagnetic nature of these nanoparticles effectively avoids the aggregation and precipitation of MACS microbeads in solution. These multicore MACS microbeads, compared with other magnetic nanoparticles, yield higher magnetic moments under the applied field of 30 Oe (Wang et al., 2014), which makes them better candidates as magnetic tags for GMR based immunoassays. Furthermore, the streptavidin homo-tetramers have an extraordinarily high binding affinity for biotin, which is among the strongest non-covalent interactions known in nature.

### One-Step Wash-Free Magnetic Bioassay

**Figure 1:**
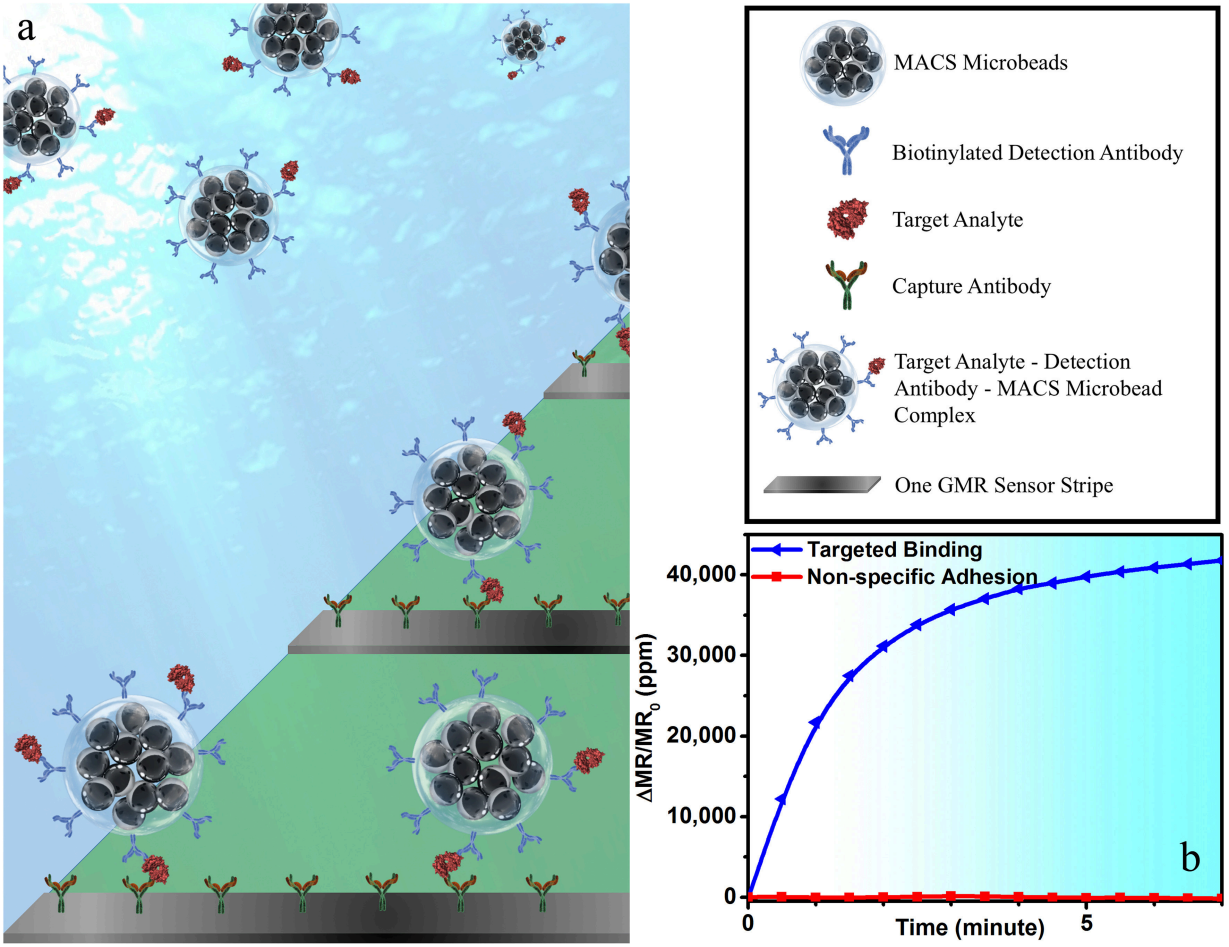
**(a)** A schematic view of one-step wash-free magnetic bioassay based on a sandwich assay structure. In each bioassay, 25 μL of MACS microbeads, 5 μL of biotinylated detection antibodies, and 20 μL of biological sample were premixed at room temperature for 1 h before transferring to the reaction well. GMR sensors were pre-coated with capture antibodies, which specifically capture the analyte-detection antibody-MACS microbead complexes. The MACS microbeads generate dipolar field upon an externally applied magnetic field. The dipolar field from those MACS microbeads captured to GMR biosensor surface change the magnetoresistance of the GMR sensor, resulting in a positive signal in **(b)**. Some of GMR biosensors were passivated with BSA to effectively block the binding of analyte-detection antibody-MACS microbead complexes to GMR biosensor surface. The red curve in **(b)** shows the signal from sensors printed with BSA, which represents the non-specific binding process.

To perform wash-free magnetic bioassay, 5 μL of 150 μg/mL of biotinylated IAV detection antibody (MAB8257B, EMD Millipore Corporation, Temecula, CA, USA, a mouse anti-influenza A monoclonal antibody specific for IAV NP) was mixed with 25 μL of MACS microbeads and 20 μL of biological sample. The mixture was placed on a rotator for 1 h at room temperature for the protein capture, and then transferred to the reaction well on the GMR chip. The capture antibody from GMR sensor surface specifically captures the target analyte-detection antibody-MACS microbead complex, forming a sandwich structure (see Figure 1a), giving rise to the positive sensor signals (see Figure 1b). This one-step immunoassay approach does not require any washing step, which effectively cuts off the assay runtime and, meanwhile, makes it possible for the measurement handled by non-technicians. Although a similar procedure has been previously demonstrated on IgG and IgM protein (Choi et al., 2016), it has never been used for real virus sample detection.

### GMR Biosensor and Detection Principle

GMR spin valve (SV) sensors were used here for the biological detection. To obtain linear MR response to the external field, the magnetization orientations of the free layer and the pinned layer are designed in a perpendicular state (Heim et al., 1994), where the resistance of the SV structure can be expressed as (Wang and Li, 2008).

$$\begin{array}{l} \text{R}={\text{R}}_{0}+\frac{1}{2}\Delta R_{max}sin\left({\Delta \theta }_{f}\right) \end{array}$$

Here, *R*_0_ is the resistance in the initial perpendicular configuration, *R*_max_ is the resistance change between the parallel and antiparallel states, and θ_*f*_ is the change of magnetization direction of the free layer (see Figure S2)

To convert the concentration of target antigens to the magnitude of magnetic signals, a sandwich structure of antibody-analyte-antibody-MNPs was immobilized on the surface of the SV sensor. Due to the specificity of the antibody-antigen reaction, only binding sites with target antigen can further build up the sandwich structure with MNPs at the top. The number of MNPs is thus proportional to the number of target antigens on the sensor surface. When exposed to an external field, only the MNPs stray field from the proximity of the sensor surface, i.e., stray field from the bound MNPs, can be picked up, while the unbound MNPs suspended in the solution will not contribute to the signal (Srinivasan et al., 2009; Li et al., 2010; Weiss et al., 2013; Wang et al., 2015). When the MNPs are added to the reaction well, each of the 21 working GMR sensors in the reaction well can generate a real-time binding curve. The sensor signal is calculated by the change in the MR after the addition of the MNPs normalized to the initial MR.

### Enzyme Linked Immunosorbent Assay (ELISA)

IAV antigen capture ELISA using monoclonal antibodies specific to influenza NP were performed as described previously (Krishna et al., 2016) Briefly, ELISA plates were coated with 100 μL of 3 μg/mL anti-influenza A monoclonal antibody (MAB8800; EMD Millipore, Temecula, CA) and incubated at 4°C overnight. After blocking the wells with 5% skim milk in PBS, 100 μL of heat-inactivated sample diluted 1:1 in sample diluent (3% BSA in PBS containing 1% IGEPAL CA-630) was added and incubated for 1 h at 37°C. Wells were washed three times with wash buffer (0.05% tween 20 in PBS) and incubated with 100 μL of 1:1000 diluted biotinylated anti-influenza A monoclonal antibody (MAB8257B; EMD Millipore, Temecula, CA) for 1 h at room temperature. Wells were washed 3 times and incubated for 30 min at room temperature with 100 μL of 1:4000 diluted streptavidin-horseradish peroxidase (HRP) (Thermo Scientific, Rockford, IL). After washing the wells three times, 100 μL of TMB peroxidase substrate (Thermo Scientific, Rockford, IL) was added and the reaction was stopped after 30 min incubation at room temperature by adding 100 μL of 1N H_2_SO_4_. The absorbance at 450 nm was measured by microtiter plate reader (Thermo Labsystems). The cut off value was calculated as mean of negative control multiplied by two. Both IAV spiked samples and nasal swab samples from the field were tested by ELISA to provide a reference for the wash-free assay.

This study was carried out in accordance with the principles of the Basel Declaration and recommendations of the Guide for the Care and Use of Laboratory Animals, University of Minnesota Institutional Animal Care and Use Committee. The protocol was approved by the University of Minnesota Institutional Animal Care and Use Committee.

### Z-Lab Diagnosis Platform and Signal Acquisition

**Figure 2:**
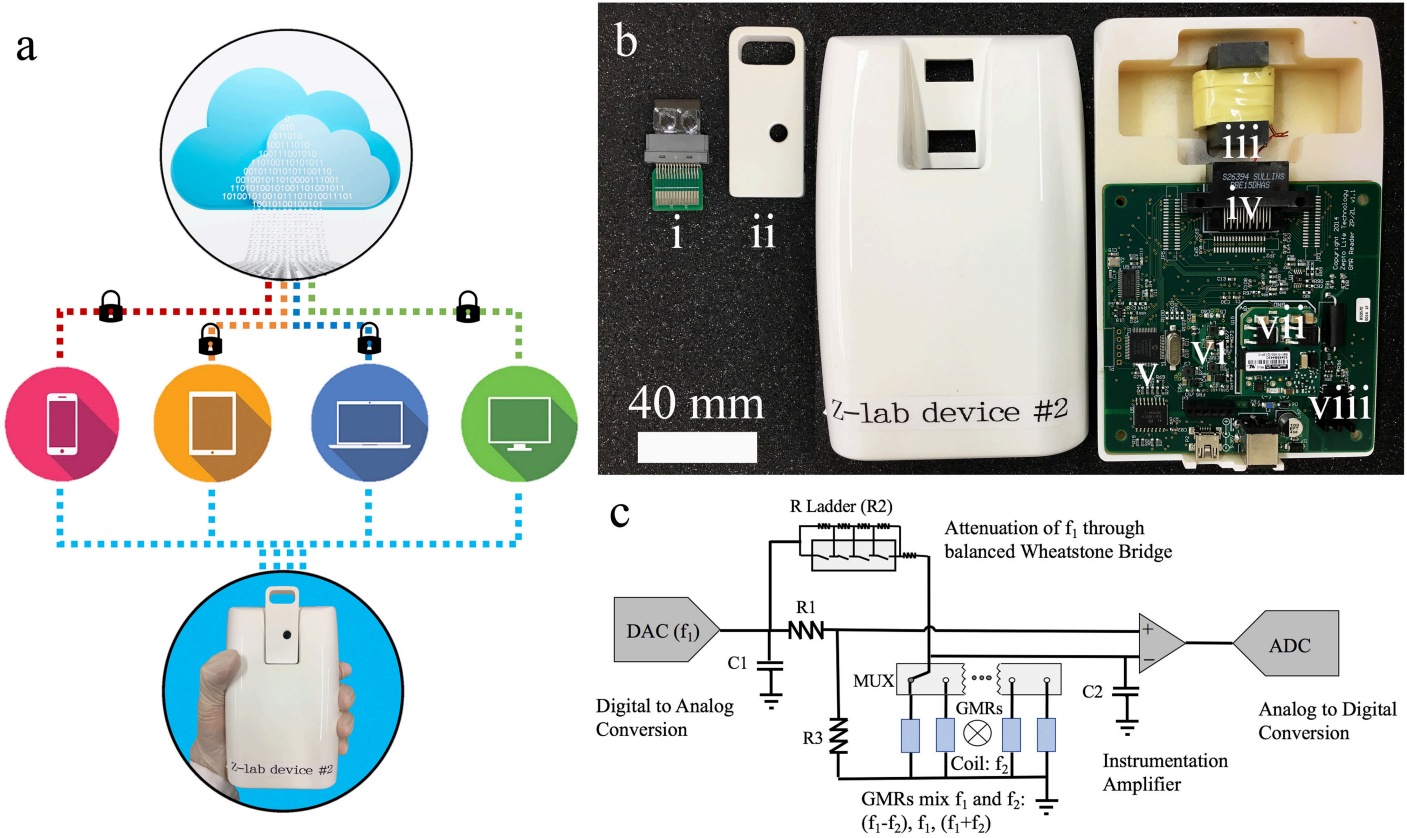
**(a)** Z-Lab handheld device communicates wirelessly with smartphones, tablets, laptops, and computers. It sends data to a secure application installed at the device form the user's end, which will be securely transmitted to the cloud storage. **(b)** Photograph of one Z-Lab diagnosis platform. (i) disposable plastic cartridge; (ii) cartridge shell; (iii) Helmholtz coil with ferrite core; (iv) card edge connector; (v) microcontroller; (vi) UART to Bluetooth and USB; (vii) power supply; (viii) current source Helmholtz coil driver. **(c)** The circuit schematic of Z-Lab.

As shown in Figure 2a, the Z-Lab diagnosis platform is handheld, portable, and can communicate wirelessly with smartphones, tablets, and computers. A Z-Lab platform consists of a reader station and a disposable cartridge. The GMR biosensor chip in the disposable cartridge is designed to detect specific biomarkers or combinations of biomarkers at low concentrations. Z-Lab can be fully integrated with modern mobile health platforms. It can wirelessly and securely transmit data to an application on a smartphone, tablet, or computer, which can be connected to a cloud-based infrastructure that can process the data in light of standard dose-response curves. Real-time and past results are available via login. Once the application/software is installed, Z-Lab is mostly automated, so that using it is only slightly more complicated than administering a rapid strep test. The circuit board design and other details about the Z-lab are shown in Figures 2b,c and reference 21.

## Results

### Detection of IAV Nucleoprotein With Wash-Free Bioassay

**Figure 3:**
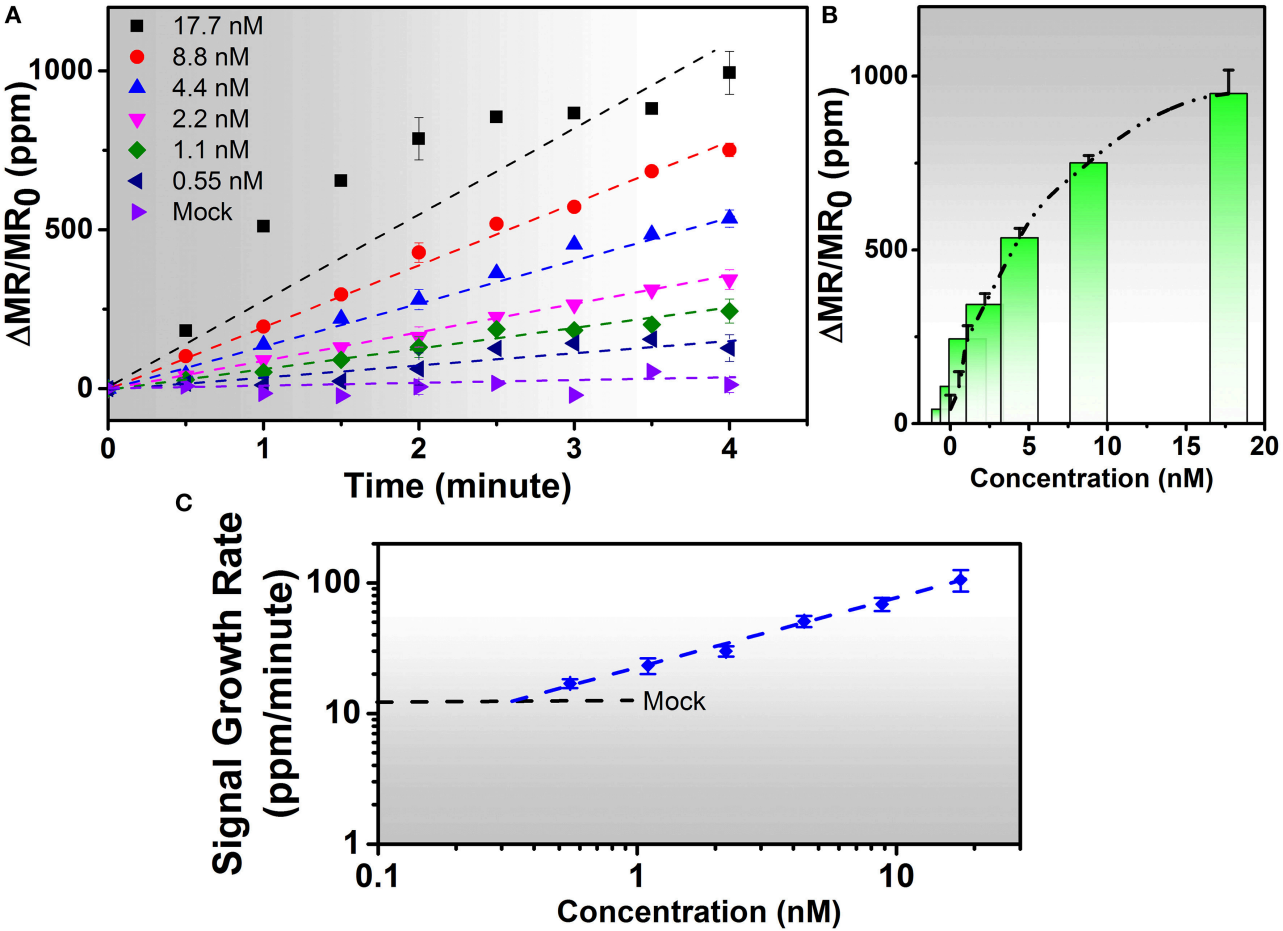
**(A)** Real-time binding curves of different concentrations of 20 μL of IAV nucleoprotein samples measured using the Z-Lab handheld device over a 4-min measurement period. As is observed from figure **(A)**, the sensor signal increases linearly during the 4-min measurement period except for 17.7 nM sample. **(B)** Averaged signals from **(A)**. The y-axis is denoted as changes in magnetoresistance (MR) normalized to initial MR in ppm (parts per million). **(C)** Calibration curves of IAV nucleoprotein bioassays. The mock level is plotted as a dashed line. Signal slopes were obtained by linear regression from *t* = 0 to *t* = 4 min to quantify the concentrations of target analytes from biological samples. Error bars in **(A,B)** indicate standard deviations of magnetoresistance signals from multiple GMR biosensors. Error bars in **(C)** indicate standard deviations of signal growth rates from multiple GMR biosensors during the time period of 4 min.

The IAV nucleoprotein sample was reconstituted in sterile PBS to make the final concentrations of 1000 ng/mL (17.7 nM), 500 ng/mL (8.8 nM), 250 ng/mL (4.4 nM), 125 ng/mL (2.2 nM), 60 ng/mL (1.1 nM), and 30 ng/mL (0.55 nM). As shown in Figure 3, the sensor signal can be characterized by the average signal as well as the signal growth rate. With the increase of IAV nucleoprotein concentration, the number of binding sites on the sensor surface also increase, resulting in a higher MR change. Since the mock sample doesn't experience specific binding process, there is no MNP on the sensor surface, resulting in negligible increase of the sensor signal. The rate of antibody-antigen reaction also changes with the analyte concentration, which is shown in Figure 3C. The lowest concentration (0.55 nM) corresponds to a signal growth rate of 16.97 ppm/min. By extrapolating the curve, the LOD for the IAV nucleoprotein detection is defined to be the concentration where the sensor signal is twice as large as the noise level of the Z-lab system, which turns out to be 0.3 nM. Compared to the traditional magnetic assays, the wash-free approach slightly lowered down the sensitivity of the detection(Wu et al., 2017a), but dramatically simplified the assay preparation process, which was crucial for a device that was designed for non-technicians to perform rapid and onsite diagnosis. Other swine-origin IAVs have also been detected and validated by ELISA in Ref. 26.

### Detection of IAV-Spiked Nasal Swab Samples With Wash-Free Bioassay

**Figure 4:**
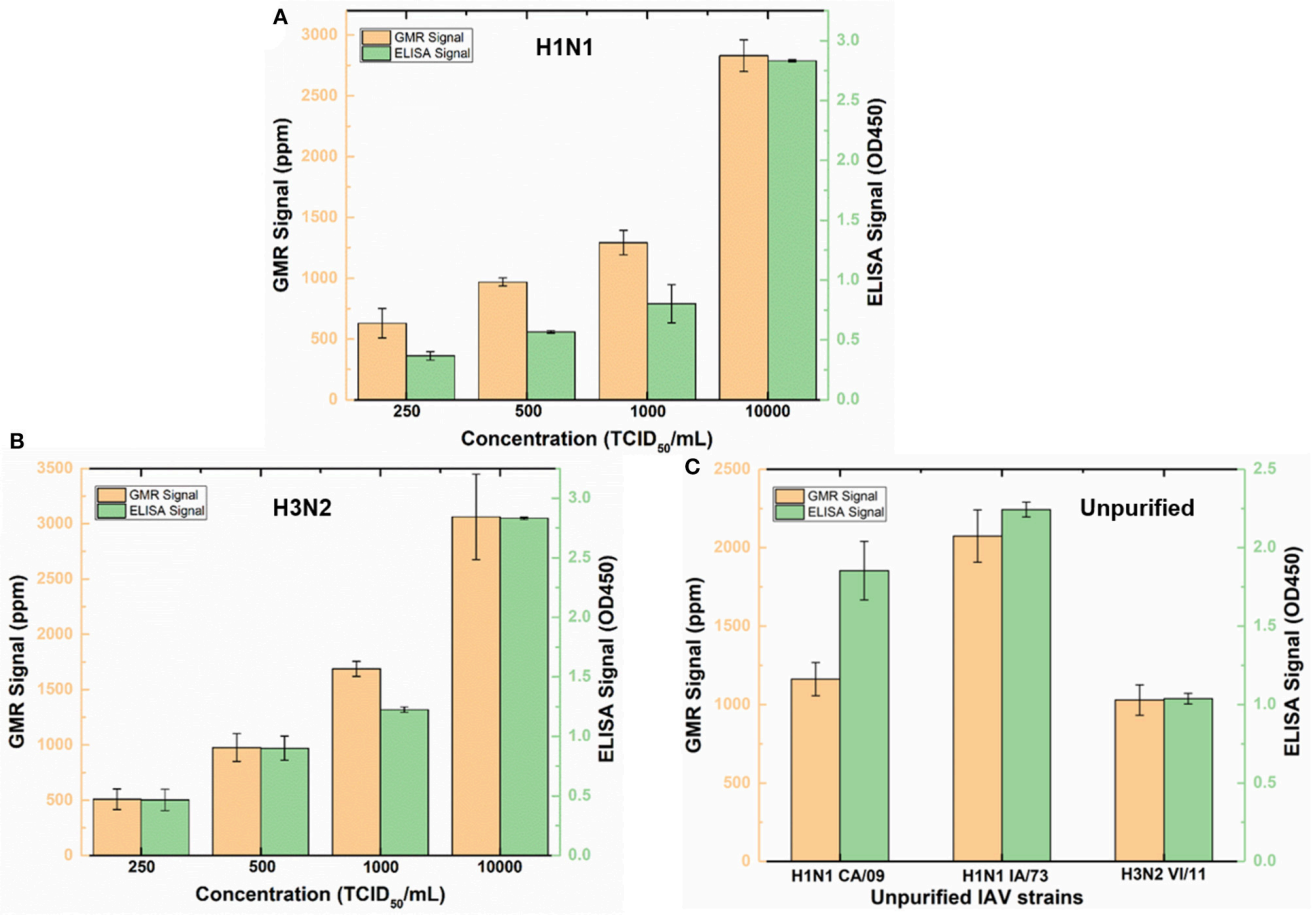
Z-lab and ELISA results for nasal swab samples spiked with 250, 500, 1,000, and 10,000 TCID_50_/mL of H1N1 CA/09 **(A)** and H3N2v **(B)** virus, and different IAV strains **(C)**. The error bars indicate the standard deviation of the signal from GMR sensors on the chip as well as from ELISA measurements.

To validate wash-free assay's ability to detect IAV in more complex matrix, nasal swab samples from IAV negative pigs were spiked with different concentrations (250, 500, 1,000, and 10,000 TCID_50_/mL) of purified IAV H3N2v or purified human pandemic H1N1 CA/09. To provide a reference, both Z-lab wash-free procedure and ELISA were performed on all the spiked samples. As shown in Figures 4A,B, the signal increases as the concentration increases for both Z-lab and ELISA. It is worth noting that a decrease of sensor signal was observed when the concentration increases from 1,000 TCID_50_/mL to 10,000 TCID_50_/mL with 5 μL of detection antibody added in the wash-free assay. At high analyte concentration, the amount of detection antibody becomes insufficient, which leads to unbound antigens suspending in the solution. The binding of antigens without magnetic tags to the capture antibody not only contribute no sensor signal, but also blocks the binding sites for other tagged antigens, leading to the decrease in the sensor signal. To provide enough number of detection antibody, 10 μL instead of 5 μL detection antibody was used in the Z-lab detection for the concentration of 10,000 TCID_50_/mL. A separate experiment was done by adding 10 μL of detection antibody to 1000 TCID_50_/mL H1N1 CA/09 sample, which resulted in a sensor signal of 1361 ppm. The sensor signal for the same sample with 5 μL of detection antibody was 1,293 ppm, which proved that the signal increase for the 10,000 TCID_50_/mL sample didn't originate from the increased amount of unbound detection antibody in the solution. The LOD of the sensor is determined by the concentration where a sensor signal is twice as large as the noise level, which is 250 TCID_50_/mL for both H1N1 CA/09 and H3N2v. To determine if Z-lab can detect other IAV strains, three different unpurified IAV isolates including swine IAV (IA/73) were randomly selected from our virus stock, diluted 1/1000 in IAV negative nasal swab samples and tested with Z-lab and ELISA (Figure 4C). All three IAV strains exhibit positive signal and similar trend in both measurements, demonstrating wash-free assay's capability of detecting different IAV strains in nasal swab samples.

### Detection of Unprocessed IAV Samples With Wash-Free Bioassay

**Figure 5:**
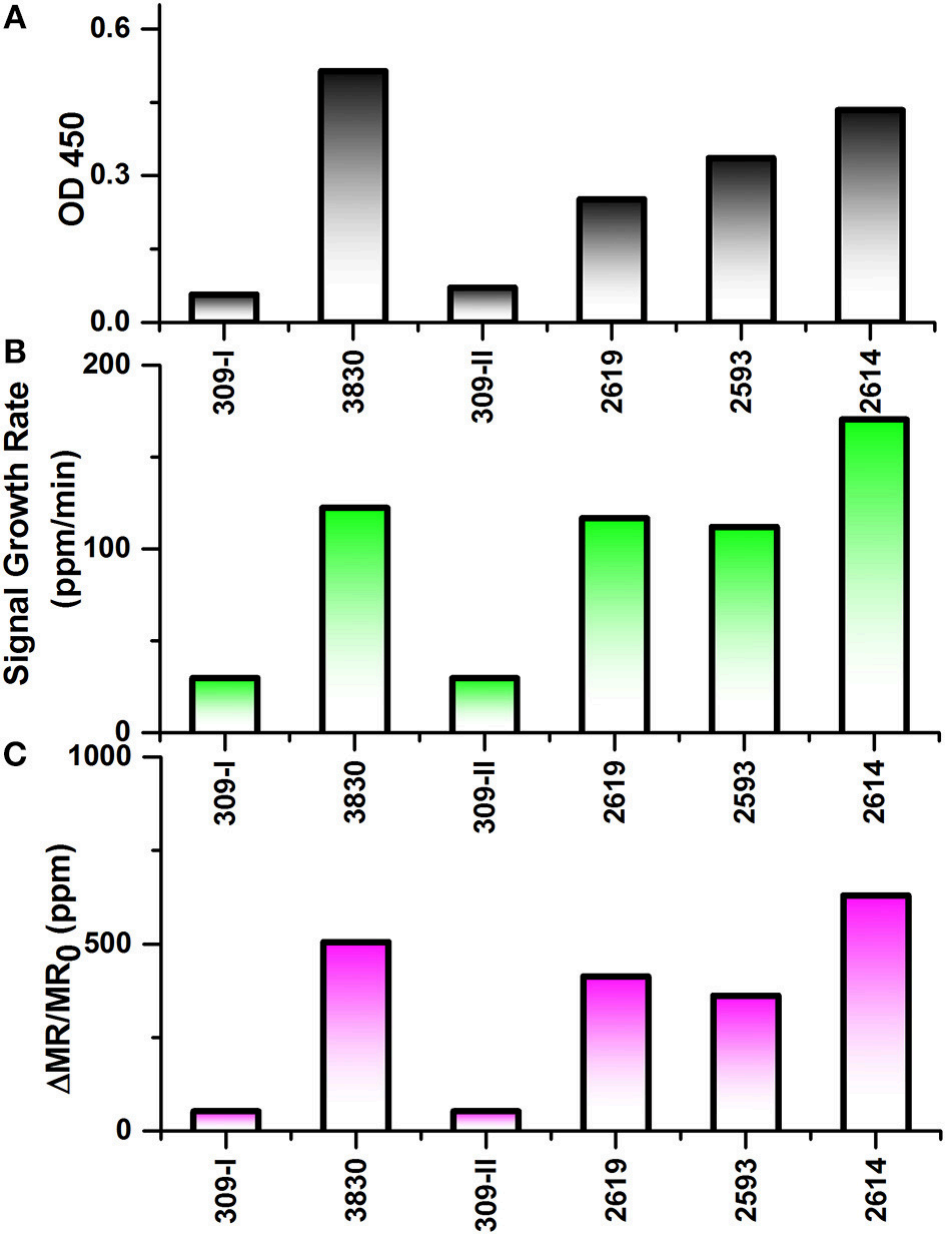
Testing results of six nasal swab samples by ELISA and Z-lab measurement. The six samples are numbered as 309-I, 3,830, 309-II, 2,619, 2,593, 2,614. **(A)** Averaged signal from 100 μL heat inactivated nasal swab samples tested by ELISA. **(B)** The signal growth rate in the first 4 min of Z-lab measurement. **(C)** Averaged signal in the first 4 min of Z-lab measurement. The error bars indicate the standard deviation of the signal from GMR sensors on the chip as well as from ELISA measurements.

As shown in Figure 5A, ELISA was carried out to compare with the performance of wash free magnetic bioassay. To determine the effect of sample matrix on the sensitivity and specificity of the assay, six nasal swab samples were tested by antigen capture ELISA. Four of these samples which are positive for influenza virus by RT-qPCR with C_T_ value ≤ 27 were also positive by ELISA with absorbance ranging from 0.25 to 0.5 (Figure 5A). However, RT-qPCR positive samples with C_T_ value >27 were negative by ELISA (data not shown).

Subsequently, the same nasal swab samples were tested with Z-lab (Figures 5B,C). Both signal growth rate and the averaged signal magnitude were used to evaluate the IAV level in the sample. Pigs 309-I and 309-II were healthy without any symptoms and other four samples were from pigs suspected to have influenza. Both the positive and negative results from Z-lab are consistent with those from ELISA, which proves the wash-free magnetic bioassay a reliable way for the “yes or no” IAV level test in the real samples taken from the field. However, the relative signal levels in the positive samples showed slightly different trend compared to the ELISA result. A possible explanation was that since the ELISA and Z-lab measurement were carried out in different labs and conditions, the IAV level might be different even for the same nasal swab sample from the same pig. The whole testing process takes 4 min, and all the bioassay preparation processes were wash-free. The wash-free bioassay has shown its own advantage due to its simple and low-cost testing process with respect to ELISA. Together with the Z-lab handheld system, this technique can perform reliable onsite “yes or no” diagnosis for the unprocessed samples before going through the expensive and time-consuming testing processes in the laboratories.

## Discussions

In this paper, we have successfully demonstrated a wash-free magnetic bioassay based on the Z-lab handheld system, which was then used for the IAV detection of nasal swab samples. By mixing the detection antibody, magnetic nanoparticles, and the antigen all together at the same time without repeated washing procedures in the traditional bio-functionalization process, this new sensing scheme managed to simplify the testing protocol with minimal sacrifice of the performance. The system showed a LOD as low as 0.3 nM for IAV nucleoprotein and < 250 TCID_50_/mL for spiked samples, with the ability to perform “yes or no” tests in unprocessed nasal swab samples.

By integrating the wash-free bioassay with the Z-lab handheld device, an accurate and efficient point-of-care device was developed, which exhibited great potential in becoming a daily routine test that can even be performed onsite in unprocessed samples by non-technicians. The testing kit consisting of GMR chip with MACS-detection antibody mixture is for one-time use and its cost will be as low as $6 each chip. The end user could simply add the biofluidic sample, detection antibody and MNP mixture into the reaction well and wait for the testing result. To realize the IAV level detection with high accuracy, the sensitivity of the system still needs to be improved, which can be achieved by increasing the stability of the sensing system and decreasing the noise level within the GMR sensors.

## Data Availability

The raw data supporting the conclusions of this manuscript will be made available by the authors, without undue reservation, to any qualified researcher.

## Ethics Statement

This study was carried out in accordance with the principles of the Basel Declaration and recommendations of the Guide for the Care and Use of Laboratory Animals, University of Minnesota Institutional Animal Care and Use Committee. The protocol was approved by the University of Minnesota Institutional Animal Care and Use Committee.

## Author Contributions

DS fabricated the GMR sensor. VK prepared the IAV nucleoprotein sample and ELISA test. DS and KW carried out the IAV detection with handheld system. TK and YF contributed to the design of the handheld device. JL characterized the MNPs. AP, MC, and JW are the supervisors and advised on the experimental design.

### Conflict of Interest Statement

JW has equity and royalty interests in, and serves on the Board of Directors, for Zepto Life Technology LLC, a company involved in the commercialization of GMR Biosensing technology. The University of Minnesota also has equity and royalty interests in Zepto Life Tech LLC. These interests have been reviewed and managed by the University of Minnesota in accordance with its Conflict of Interest policies. The remaining authors declare that the research was conducted in the absence of any commercial or financial relationships that could be construed as a potential conflict of interest.

## References

[B1] Amano Y. Cheng Q. (2005). Detection of influenza virus: traditional approaches and development of biosensors. Anal. Bioanal. Chem. 381, 156–164. 10.1007/s00216-004-2927-0 15592819

[B2] Baselt D. R. Lee G. U. Natesan M. Metzger S. W. Sheehan P. E. Colton R. J. (1998). A biosensor based on magnetoresistance technology. Biosens. Bioelectron. 13, 731–739. 10.1016/S0956-5663(98)00037-2 9828367

[B3] Cardoso S. Leitao D. C. Dias T. M. Valadeiro J. Silva M. D. Chicharo A. (2017). Challenges and trends in magnetic sensor integration with microfluidics for biomedical applications. J. Phys. D Appl. Phys. 50:213001 10.1088/1361-6463/aa66ec

[B4] Chartrand C. Leeflang M. M. Minion J. Brewer T. Pai M. (2012). Accuracy of rapid influenza diagnostic tests a meta-analysis. Ann. Intern. Med. 156, 500–U80. 10.7326/0003-4819-156-7-201204030-00403 22371850

[B5] Chen Y. Cui D. Zheng S. Yang S. Tong J. Yang D.. (2011). Simultaneous detection of influenza A, influenza B, and respiratory syncytial viruses and subtyping of influenza A H3N2 virus and H1N1 (2009) virus by multiplex real-time PCR. J. Clin. Microbiol. 49, 1653–1656. 10.1128/JCM.02184-10 21270233 PMC3122825

[B6] Choi J. Gani A. W. Bechstein D. J. B. Lee J. R. Utz P. J. Wang S. X. (2016). Portable, one-step, and rapid GMR biosensor platform with smartphone interface. Biosens. Bioelectron. 85, 1–7. 10.1016/j.bios.2016.04.046 27148826

[B7] Deepa I. (1971). A revised system of nomenclature for influenza viruses. Bull. World Health Organ. 45, 119–124. 5316848 PMC2427881

[B8] Dias T. M. Cardoso F. A. Martins S. A. M. Martins V. C. Cardoso S. Gaspar J. F. (2016). Implementing a strategy for on-chip detection of cell-free DNA fragments using GMR sensors: a translational application in cancer diagnostics using ALU elements. Anal. Methods 8, 119–128. 10.1039/C5AY01587A

[B9] Ellis J. S. Zambon M. C. (2002). Molecular diagnosis of influenza. Rev. Med. Virol. 12, 375–389. 10.1002/rmv.370 12410529

[B10] Gaster R. S. Xu L. Han S.-J. Wilson R. J. Hall D. A. Osterfeld S. J.. (2011). Quantification of protein interactions and solution transport using high-density GMR sensor arrays. Nat. Nanotechnol. 6, 314–320. 10.1038/nnano.2011.45 21478869 PMC3089684

[B11] Heim D. E. Fontana R. E. Tsang C. Speriosu V. S. Gurney B. A. Williams M. L. (1994). Design and operation of spin-valve sensors. IEEE Trans. Magn. 30, 316–321. 10.1109/20.312279

[B12] Kida H. Ito T. Yasuda J. Shimizu Y. Itakura C. Shortridge K. F.. (1994). Potential for transmission of avian influenza-viruses to pigs. J. Gen. Virol. 75, 2183–2188. 10.1099/0022-1317-75-9-2183 8077918

[B13] Krishna V. D. Wu K. Perez A. M. Wang J.-P. (2016). Giant Magnetoresistance-based biosensor for detection of influenza A virus. Front. Microbiol. 7:400. 10.3389/fmicb.2016.00400 27065967 PMC4809872

[B14] Lee B. W. Bey R. F. Baarsch M. J. Simonson R. R. (1993). ELISA Method for detection of influenza A infection in swine. J. Vet. Diagnostic Invest. 5, 510–515. 10.1177/104063879300500402 8286447

[B15] Leuwerke B. Kitikoon P. Evans R. Thacker E. (2008). Comparison of three serological assays to determine the cross-reactivity of antibodies from eight genetically diverse U.S. Swine Influenza Viruses. J. Vet. Diagnostic Invest. 20, 426–432. 10.1177/104063870802000403 18599846

[B16] Li Y. P. Srinivasan B. Jing Y. Yao X. F. Hugger M. A. Wang J. P.. (2010). Nanomagnetic competition assay for low-abundance protein biomarker quantification in unprocessed human sera. J. Am. Chem. Soc. 132, 4388–4392. 10.1021/ja910406a 20192199

[B17] Merckx J. Wali R. Schiller I. Caya C. Gore G. C. Chartrand C.. (2017). Diagnostic accuracy of novel and traditional rapid tests for influenza infection compared with reverse transcriptase polymerase chain reaction a systematic review and meta-analysis. Ann. Internal Med. 167, 394–409. 10.7326/M17-0848 28869986

[B18] Miller E. Hoschler K. Hardelid P. Stanford E. Andrews N. Zambon M. (2010). Incidence of 2009 pandemic influenza A H1N1 infection in England: a cross-sectional serological study. Lancet 375, 1100–1108. 10.1016/S0140-6736(09)62126-7 20096450

[B19] Payungporn S. Chutinimitkul S. Chaisingh A. Damrongwantanapokin S. Buranathai C. Amonsin A.. (2006). Single step multiplex real-time RT-PCR for H5N1 influenza A virus detection. J. Virol. Methods 131, 143–147. 10.1016/j.jviromet.2005.08.004 16183140

[B20] Reiss G. Brueckl H. Huetten A. Schotter J. Brzeska M. Panhorst M. (2005). Magnetoresistive sensors and magnetic nanoparticles for biotechnology. J. Mater. Res. 20, 3294–3302. 10.1557/jmr.2005.0409

[B21] Ren H. Jin Y. Hu M. Zhou J. Song T. Huang Z.. (2016). Ecological dynamics of influenza A viruses: cross-species transmission and global migration. Sci. Rep. 6:36839. 10.1038/srep36839 27827462 PMC5101809

[B22] Rizzi G. Østerberg F. W. Dufva M. Hansen M. F. (2014). Magnetoresistive sensor for real-time single nucleotide polymorphism genotyping. Biosens. Bioelectron. 52, 445–451. 10.1016/j.bios.2013.09.026 24094523

[B23] Schotter J. Kamp P. B. Becker A. Pühler A. Reiss G. Brückl H. (2004). Comparison of a prototype magnetoresistive biosensor to standard fluorescent DNA detection. Biosens. Bioelectron. 19, 1149–1156. 10.1016/j.bios.2003.11.007 15046745

[B24] Srinivasan B. Li Y. P. Jing Y. Xu Y. H. Yao X. F. Xing C. G.. (2009). A detection system based on giant magnetoresistive sensors and high-moment magnetic nanoparticles demonstrates zeptomole sensitivity: potential for personalized medicine. Angew. Chem. Int. Edn. 48, 2764–2767. 10.1002/anie.200806266 19288507

[B25] Tondra M. Porter M. Lipert R. J. (2000). Model for detection of immobilized superparamagnetic nanosphere assay labels using giant magnetoresistive sensors. J. Vac. Sci. Technol. A-Vac. Surf. Films 18, 1125–1129. 10.1116/1.582476

[B26] Townsend M. B. Dawson E. D. Mehlmann M. Smagala J. A. Dankbar D. M. Moore C. L.. (2006). Experimental evaluation of the fluchip diagnostic microarray for influenza virus surveillance. J. Clin. Microbiol. 44, 2863–2871. 10.1128/JCM.00134-06 16891504 PMC1594652

[B27] Wang H. M. Deng J. K. Tang Y. W. (2018). Profile of the Alere i Influenza A & B assay: a pioneering molecular point-of-care test. Expert Rev. Mol. Diagn. 18, 403–409. 10.1080/14737159.2018.1466703 29688086 PMC6153442

[B28] Wang S. X. Li G. (2008). Advances in giant magnetoresistance biosensors with magnetic nanoparticle tags: Review and outlook. IEEE Trans. Magn. 44, 1687–1702. 10.1109/TMAG.2008.920962

[B29] Wang W. Wang Y. Tu L. Feng Y. Klein T. Wang J.-P. (2014). Magnetoresistive performance and comparison of supermagnetic nanoparticles on giant magnetoresistive sensor-based detection system. Sci. Rep. 4:5716. 10.1038/srep05716 25043673 PMC4104391

[B30] Wang W. Wang Y. Tu L. Klein T. Feng Y. L. Wang J. P. (2013). Surface modification for protein and DNA immobilization onto GMR biosensor. IEEE Trans. Magn. 49, 296–299. 10.1109/TMAG.2012.2224327

[B31] Wang Y. Wang W. Yu L. N. Tu L. Feng Y. L. Klein T.. (2015). Giant magnetoresistive-based biosensing probe station system for multiplex protein assays. Biosens. Bioelectron. 70, 61–68. 10.1016/j.bios.2015.03.011 25794959

[B32] Weiss R. Mattheis R. Reiss G. (2013). Advanced giant magnetoresistance technology for measurement applications. Meas. Sci. Technol. 24, 082001 10.1088/0957-0233/24/8/082001

[B33] Wu K. Klein T. Krishna V. D. Su D. Q. Perez A. M. Wang J. P. (2017a). Portable GMR handheld platform for the detection of influenza A virus. Acs Sens. 2, 1594–1601. 10.1021/acssensors.7b00432 29068663

[B34] Wu K. Schliep K. Zhang X. Liu J. Ma B. Wang J. P. (2017b). Characterizing physical properties of superparamagnetic nanoparticles in liquid phase using Brownian relaxation. Small 13. 10.1002/smll.201604135 28374941

[B35] Zhi X. Liu Q. S. Zhang X. Zhang Y. X. Feng J. Cui D. X. (2012). Quick genotyping detection of HBV by giant magnetoresistive biochip combined with PCR and line probe assay. Lab Chip 12, 741–745. 10.1039/c2lc20949g 22222368

