# Supplementary material for: Detection of Influenza a Virus in Swine Nasal Swab Samples With a Wash-Free Magnetic Bioassay and a Handheld Giant Magnetoresistance Sensing System
Source: Front Microbiol. 2019 May 21;10:1077. doi: 10.3389/fmicb.2019.01077 (PMC6536586; doi:10.3389/fmicb.2019.01077)
Supplement: Supplementary file 1 [file Data_Sheet_1.docx]

Supplementary Material

**Detection of Influenza A Virus in Nasal Swab Samples With A Wash-Free Magnetic Bioassay and A Handheld Giant Magnetoresistance Sensing System**

**Diqing Su^†, ⊥^, Kai Wu^‡, ⊥^, Venkatramana D. Krishna^§, ⊥^, Todd Klein^‡^, Jinming Liu^‡^, Yinglong Feng^‡^, Andres M. Perez^§^, Maxim C-J Cheeran^§, *^ and Jian-Ping Wang^†, ‡, *^**

*** Correspondence:** Jian-Ping Wang: [jpwang@umn.edu](mailto:jpwang@umn.edu), Maxim C-J Cheeran: [cheeran@umn.edu](mailto:cheeran@umn.edu)

**Configuration and MR Curve of GMR Nanosensor Array**

**
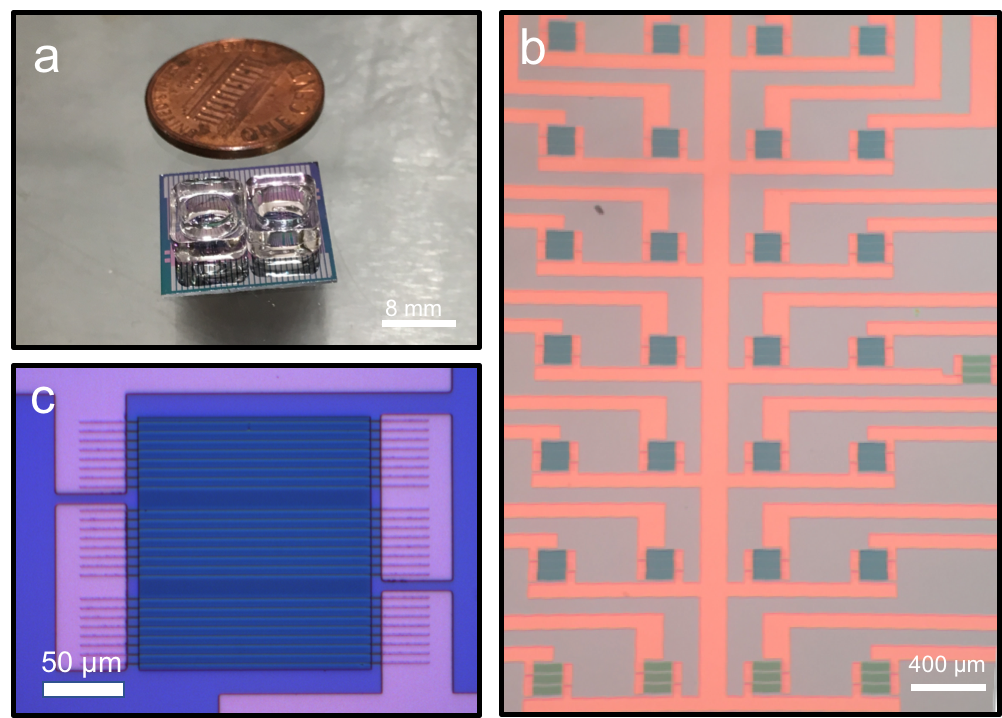
**

**Figure S1.** GMR sensor arrays. (a)There are two GMR sensor arrays on each chip, which can accommodate two reaction wells. (b) Each sensor array is made up of 29 GMR sensors. All of the sensors are connected to the electrodes on the edge of the chip, which provides facilities for subsequent signal acquisition. (c) An individual GMR sensor consists of 24 strips, which are 150 μm long and 750 nm wide. The stripes are divided into 5 groups connected in series and each of the group has 8 stripes connected in parallel. This configuration yields a resistance of ~2000 Ohms for one GMR sensor.


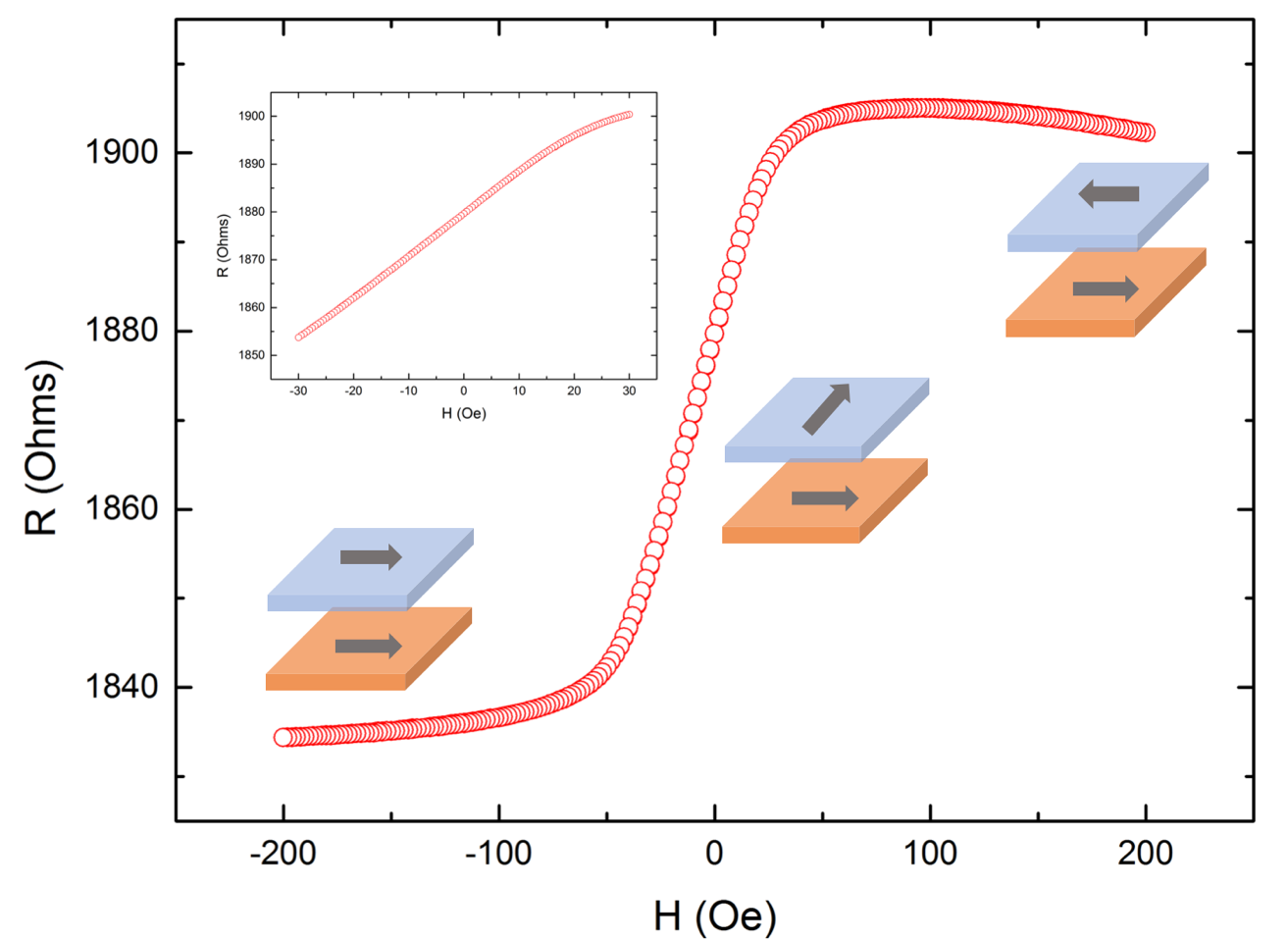


**Figure S2. MR curve of a spin valve sensor.** When the external magnetic field change from -200 Oe to 200 Oe, the magnetization configuration of free layer and pinned layer will change from antiparallel state to parallel state, which results in the decrease in the resistance of the structure. The MR of the sensor shown in this Figure is 3.7%, which is defined as ${(R}_{max}-R_{min})/R_{min}\times100\%$. The GMR stack and the shape of the sensor stripe are designed so that the MR response can achieve best linearity and sensitivity fo the detection of the magnetic field from the magnetic nanoparticles. During detection, a bias magnetic field of -30 Oe ~30 Oe is applied in the plane of sensor surface. The magnetoresistance of the sensor in this region is 2.5%. During detection, the MNPs are magnetized and can be viewed as a magnetic dipole, whose magnetic field can be picked up by the GMR sensor below, resulting in the change of the MR value of the sensor.

**Surface Chemistry of Biofunctionalization**

**
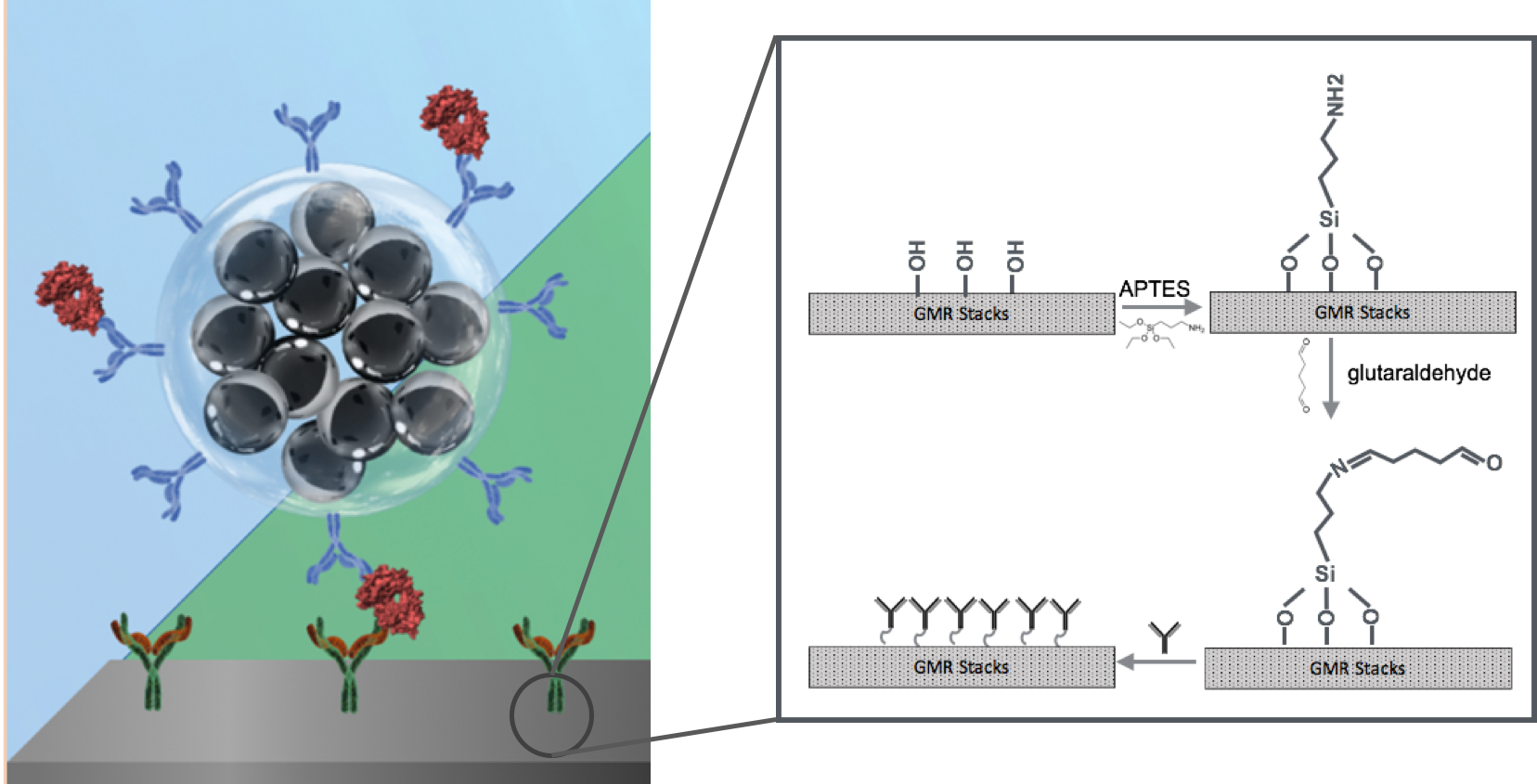
**

**Figure S3. Immobilization process of capture antibody.** The surface of the GMR sensors are covered with a thin SiO_2_ layer. Amino groups are introduced to the sensor surface through reaction between the free hydroxyl groups on the SiO_2_ and APTES. Subsequently, the sensor area is covered with glutaraldehyde via the aldehyde-amino reaction. The capture antibody is then immobilized on the other aldehyde group on the glutaraldehyde. The antigen and detection antibody are introduced by the specific antibody-antigen reaction, while the MNPs are bound to the detection antibody through the reaction between the biotin on the detection antibody and the streptavidin on the MNPs.


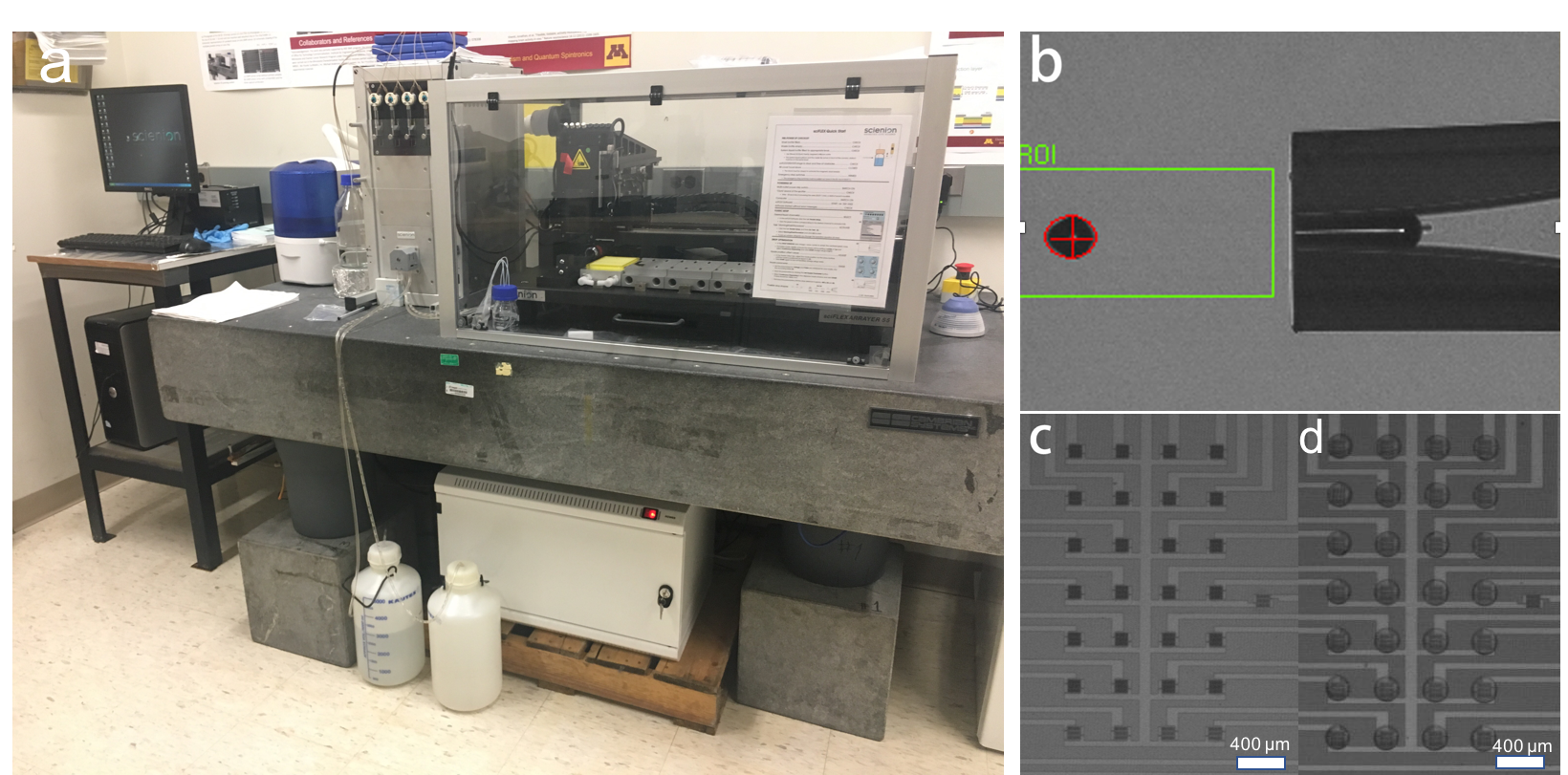


**Figure S4. Printing of capture antibody.** Photographs of (a) sci-FLEXARRAYER S5 Liquid Dispensing System, (b) a single drop from the nozzle, which, with a volume of 400 pL, and the sensing area before (c) and after (d) the printing of capture antibody.


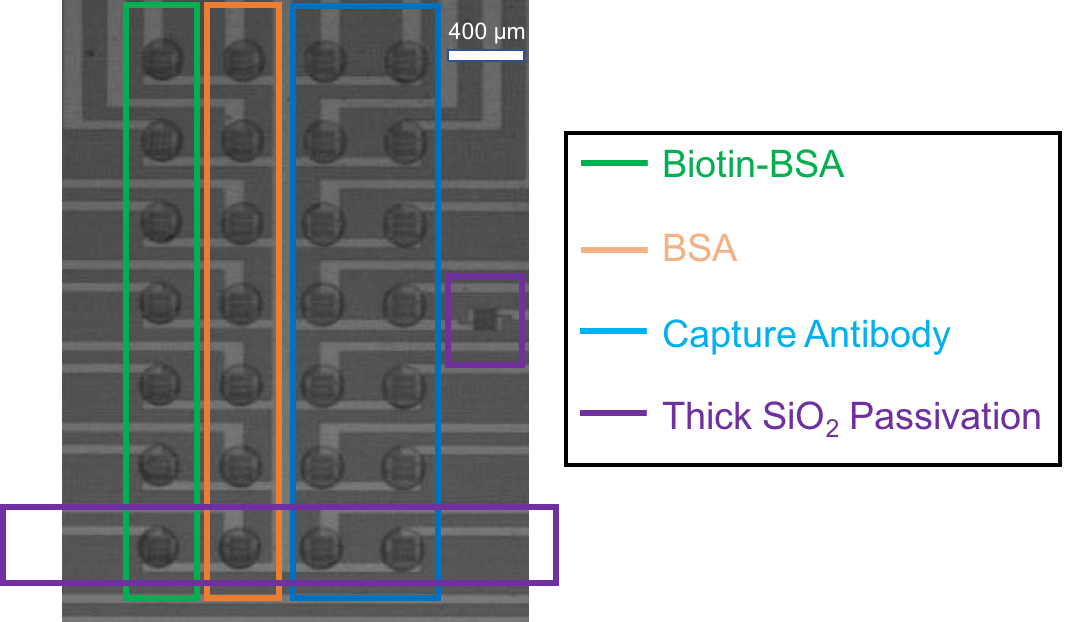


**Figure S5.** The first column of sensors is printed with biotin-BSA. Since biotin-streptavidin interaction has the highest binding affinity in nature compared to any other antibody-antigen reactions, this column of sensors will have the largest signal, which serves as the positive control group. The second column of sensors is spotted with BSA, which will not bind specifically to the target antigen and serves as the negative control group. The other two columns of the sensors are spotted with capture antibody for the biomarker detection. Additionally, the last row of the sensors as well as the single sensor on the right are coated with thick SiO_2_ passivation layer to serve as another negative control group. The large distance between the MNPs and sensor surface will yield zero signal on these sensors.
